# Surgical site infections following caesarean sections at Emirati teaching hospital: Incidence and implicated factors

**DOI:** 10.1038/s41598-020-75582-9

**Published:** 2020-10-30

**Authors:** Munther S. Alnajjar, Dalia A. Alashker

**Affiliations:** 1 Department of Biopharmaceutics & Clinical Pharmacy, College of Pharmacy, Al-Ahliyya Amman University, P. O. Box 19328, Amman, Jordan; 2 Department of Clinical Pharmacy, College of Pharmacy, Al Ain University, Al Ain, United Arab Emirates; 3 Pharmacy Department, Al Ain Hospital, Al Ain, United Arab Emirates

**Keywords:** Microbiology, Diseases, Risk factors

## Abstract

The rate of delivery by caesarean sections is increasing globally and, therefore, the incidence of post-caesarean surgical site infections (SSIs) is probably also going to rise. The aim of the present study was to determine the incidence of SSIs after caesarean operations and to explore the factors associated with an increased risk of post-caesarean SSIs. A retrospective study was performed to assess all women who underwent caesarean sections from January 2016 to December 2017 at Al Ain Hospital in the United Arab Emirates (UAE). Backward multivariate logistic regression analysis was utilized to specify the variables that were significantly and independently connected with the development of post-caesarean SSIs. In total, 807 women underwent caesarean deliveries at the study site hospital during the two-year study period (January 2016–December 2017). Post-operative SSI was detected in 11 (1.4%) of the women who underwent caesarean operations. Of these, 11 (100%) women were diagnosed post-discharge, within 30 days after the date of the surgery. Multivariate logistic regression analysis showed that increased gestational age (*P* = 0.045) was significantly and independently associated with the development of post-caesarean SSI. Increased gestational age was found to be an independent predictor of post-caesarean SSIs. This identified risk factor should inform targeted health care policies to reduce the rate of SSIs.

## Introduction

Caesarean sections are a major surgery performed by obstetricians across the world^1^. During 2000–2015, worldwide average annual caesarean delivery use increased by 3.7%. In 2015, caesarean section utilization ranged widely across nine regions, from 4.1% (3.6–4.6%) in western and central Africa to 44.3% (41.3–47.4%) in the Latin America and Caribbean region^2^. Almost half of the childbirths studied during the period 2013–2014 in Poland, were births by caesarean section. But in many countries, it is much less, such as 30% (in the US), 14.7% (Finland) and 16.2% (Sweden)^3^. In the United Arab Emirates (UAE), the rate also increased; according to the WHO World Statistics Report published in 2015, slightly over a quarter of all births in the UAE were caesarean sections, which is almost double the global recommended rate (10% to 15% )^4^. Although they can save lives, caesarean sections are often performed without therapeutic need, putting women and their infants at danger of short- and long-term health issues. Caesarean sections might be essential when vaginal delivery represents a danger to the mother or child, for instance, because of prolonged labour, foetal trouble, or because the infant is in an abnormal position. In a study conducted in Italy to analyse the obstetric complications, it was found that 60% of deliveries were caesarean sections and 33.3% were performed because of previous bowel surgery^5^. Nonetheless, caesarean sections can lead to many significant complications; one of these critical complications is surgical site infections (SSIs)^4^. Recuperation after caesarean section can be distressing for low-income women, who often develop a surgical site infection, especially after early hospital discharge. These women typically have little expertise in dealing with wounds and have little help from anyone else at home^6^. Additionally, having repeated and/or urgent caesarean sections were found also associated with a 5.0-fold increases risk of bladder injury after accounting for other risk factors^7^.

According to the CDC in 2017, SSIs are an important cause of healthcare-associated infections (HCAIs). Of note, 14% of infections are SSIs and almost 5% of patients who have undergone an operation developed an SSI^8,9^. However, SSIs are usually underestimated in incidence studies, since many of them occur after the patient is discharged from the hospital^10–13^. According to the CDC, SSIs are the second most common infectious complication following caesarean section, after urinary tract infections (UTIs). Their incidence ranges from 3 to 15%. The frequency of caesarean section SSIs was found to be 12% in Nigeria, 29.38% in Oman, and 9.6% in Thailand^14,15^. Other studies have found that the caesarean section SSI frequency in England was 51% in the obese population, 13.5% in the United States, and 10% in the general population of Australia^16^.

Surgical site infection remains a serious source of morbidity and mortality, particularly in developing countries. SSIs are defined as infections that are observed 30 days after surgery and affect the incision or profound tissue in the surgical site^9,17,18^. SSIs can range from a mild wound discharge without complications to life-threatening sepsis^10,13,19^. According to Menacker and Hamilton (2010), the risk of complications for the mother and baby due to a caesarean procedure also potentiates the risk for increased costs. Readmissions due to SSIs have been estimated to cost approximately $50,000^20^. Patients with SSIs don't generally get readmitted; however, they may use a critical number of resources, including doctors’ offices or emergency division visits, which in turn increase health costs^21^.

Several studies have examined the factors that increase the chance of performing a caesarean and increase the risk of complications. The risk factors identified by most studies include obesity, advanced maternal age, diabetes, poor prenatal care, immunosuppressive disorders, chorioamnionitis, a previous caesarean delivery, certain medications like steroids, a lack of pre-incision antimicrobial care, excessive blood loss during labour, delivery, or surgery, and lengthy labour and surgery^22–26^. Many studies have demonstrated that antimicrobial prophylaxis reduces post-operative surgical infection by reducing the likelihood of resident bacteria overcoming the immune system^27^. Lacking pre-operative antibiotic prophylaxis, increases American Society Anaesthesiologist ASA score and extended surgery duration were found potential predictors for acquisition of post-surgical SSI in Polish Hospitals after caesarean delivery^28^. Re-hospitalizations due to surgical site infections were significantly more often recorded among women living outside big cities and consequently giving birth in primary referral hospitals with a low level of healthcare quality^29^.

However, no study has been done so far on the incidence and risk factors of post-caesarean delivery SSI in large referral teaching hospitals in the UAE. This study was done to determine the incidence of and identify the risk factors associated with post-caesarean SSIs across a tertiary care and teaching hospital in the UAE. Understanding the risk factors associated with SSIs after caesarean operations will help to increase healthcare professionals’ awareness of this issue in the future. We hypothesized that particular patient and maternal-related risk factors would increase the incidence of SSIs following caesarean sections in Al Ain Hospital (AAH). This study aimed at investigating the factors that may be linked to surgical site infections following caesarean procedures in AAH, Al Ain, UAE.

## Methods

### Study setting

The study was conducted in AAH, a tertiary care and teaching hospital in the UAE. It is a leader in multidisciplinary outpatient diagnostics and treatments in the region. AAH has over 35 specialist departments with more than 300 doctors in total. The hospital consists of 402 beds and is an acute and emergency hospital. It serves the community of Al Ain and its region, with more than 20,000 patients and 320,000 patients in their outpatient clinics annually.

### Study design and study period

The study was a retrospective study assessing the risk factors for SSIs following caesarean sections among women at AAH undergoing caesarean procedures between January 2016 and December 2017. The analysis was based only on women who were followed up for 30 days after the operation date. The patients’ electronic medical records were examined and data were collected on potential risk factors. In this study, the independent variables included the age of the patient, BMI, gestational age, anaesthesia type and duration, duration of the operation, mode of delivery, length of stay at hospital, number of caesarean operations, administration of prophylactic antibiotics, timing of antibiotic prophylaxis, and the presence of diabetes. All available wound culture results for the included patients were retrieved from the microbiology laboratory computer system of the hospital and positive cultures were recorded; at AAH, as part of routine practice, wound cultures are usually ordered by the attending physician when upon clinical examination of the wound signs of infection are observed. However, the method used for taking samples included only swabs. The dependent variable was the identification of the post-caesarean SSI (yes/no). Backward logistic regression analyses were carried out to determine the risk factors independently associated with the development of SSIs following caesarean procedures.

### Patients sampling method

The patients were chosen for this study using a convenience sample including all cases over a two-year study period. Sequential sampling of all women who had caesarean delivery was done. This method was chosen because all women who had undergone caesarean delivery were at risk of the developing SSIs. A list of all patients who underwent caesarean delivery at the AAH between January 2016 and December 2017 was generated using ICD-9 and ICD-10 codes. The medical records of patients with at least one of these codes were reviewed to determine whether they met the primary interest outcome criteria. Determination of SSI case required both the presence of diagnostic code and one of the conditions reported in the medical records. To detect SSIs within 30-days post-discharge, follow-up period, patient medical/nursing records, and surgery clinic patient records were reviewed for all women who were readmitted or transferred to an obstetrics and gynaecology ward or clinic following a caesarean procedure at AAH (between January 2016 and December 2017). The documentation was reviewed as follows: (1) admission, readmission, emergency department, and operation room logs; (2) patient charts for signs and symptoms of SSI; (3) lab, imaging, and other diagnostic test reports; (4) clinician notes; (5) ICD-10-CM and ICD-9 infection diagnosis codes. Additionally, to monitor and manage any detected SSIs, the patients were routinely instructed by hospital midwives (and patient counselling personnel) to judge the presence of SSIs and to arrange immediately for a confirmatory diagnostic examination at AAH. Additionally, as a part of routine practice, a nurse from the hospital infection control team was allocated to respond to any clarifications or queries requested regarding any wound signs of SSI appeared 30 days after operation date, as well as to arrange for a follow-up visit, into the hospital outpatient clinic, or for rehospitalisation within obstetrics and gynaecology ward (if required).

### Study inclusion and exclusion criteria

The inclusion criteria included all women who underwent caesarean delivery during the study period and women have been readmitted or transferred to an obstetrics and gynaecology inpatient ward or outpatient clinic for an SSI after a caesarean procedure at AAH. The following patients were excluded: normal deliveries, women who had a caesarean section done elsewhere and having infection from another source, e.g., respiratory.

### Case definitions

The definitions and criteria of CDC for surveillance of caesarean SSI, were applied as follows:

- A superficial incisional post-caesarean SSI.
  Superficial infection episodes were detected via applying the following criteria: if the infection appeared within 30 days of the caesarean procedure; only incision tissue was infected, infection involved skin and subcutaneous layers; and at least one of the following listed events/conditions was present: (i) purulent exudates excreted from the first cell layers of skin (superficial tissue); (ii) microbes recovered from superficial skin tissue; (iii) presence of pain, swelling, heat, or redness at the wound incision site; or (iv) superficial infection was diagnosed clinically by surgeon or attending physician following caesarean delivery^17,30^.
- A deep incisional post-caesarean SSI.
  The following criteria were used to confirm each deep incision case: for patients who had no implant, infection incidence appeared within 30 days of the procedure (or identified within 90 days postoperatively if an implant was left in place); development of infection within the deep incision tissue, e.g. muscle tissue layer; and at least one of the following events: (1) yellowish excretions being produced from deep tissue of skin incision site; (2) dehisces of incision site deep layers, combined with either fever (> 38 °C) or tenderness; (3) appearance of abscess within deep layers of the surgical site; or (iv) deep SSI was detected clinically by obstetrician or surgeon^17,30^.
- Organ space post-caesarean SSI.
  The following conditions were met when defining an organ space SSI: infection was documented throughout the 30 days after undergoing the caesarean procedure; the case related to any body site (e.g. arterial/venous infection, endometritis, vaginal cuff infection, ovaries and uterus), rather than superficial skin or muscle layers of the incision site; at least one of the following conditions was observed: (1) release of purulent drainage from wound localised within the above specified organs; (2) recovery of bacteria from tissue of fluid in the infected organ; (3) detection of abscess within the infected organ; or (4) organ space infection was diagnosed by surgeon or attending physician, post-operation^17,30^.

### Statistical analyses

The demographic and maternal characteristics of all patients who underwent a caesarean section were determined using descriptive statistics (e.g., categorical variables reported as proportions and continuous variables reported as means). Several tests were used to identify individual relationships between the data collected on potential risk factors and the development of post-caesarean SSIs: (1) the Mann–Whitney U-test was employed with non-normally distributed scale variables; and (2) Pearson’s Chi-square test was used with categorical variables (Fisher’s exact test was used when the data did not fit the criteria of the Chi-square test). Following this, a backward logistic regression analysis was carried out (all variables that had *P* values of < 0.2 or that were clinically important for univariate analysis were included) to identify significant independent risk factors for SSIs. The odds ratio (OR) approach was used to express the extent of the increased or decreased odds ratio of developing an SSI (with *P* ≤ 0.05 deemed statistically significant). All the statistical analysis procedures were performed using the Statistical Package for Social Sciences, Windows Version 25.0 (SPSS, Chicago).

### Ethical approval

All procedures performed in this study involving human participants were in accordance with the ethical standards of the institutional and/or national research committees and with the 1964 Helsinki declaration and its later amendments or comparable ethical standards. For the purposes of conducting this study, governance approvals were obtained from the Research Ethical Committee in AAH, Emirate of Abu Dhabi, UAE (Reference No: AAHEC-04-18-093). For this type of study (retrospective in design), formal individual patient consent was not required by AAH Research and Ethics Governance Committee.

## Results

### Incidence rate of post-caesarean SSI

Out of a total of 4650 childbirths occurred over the two-year study period (Jan 2016–Dec 2017) at AAH, 807 (17.4%) women had caesarean sections. Of these 807, a total of 796 (98.6%) women had no SSI, and 17 (2.1%) women developed SSIs according to the primary definition. Out of these 17 cases, 3 (0.38%) patients were excluded as their caesarean procedures were performed elsewhere, and further 3 (0.38%) post-caesarean SSIs were excluded as the infection was originated from another source (specifically from community acquired pneumonia). It was found that 11 (100.0%) women were diagnosed post-discharge, within 30 days after the operation date, and none (0.0%) were detected during the hospital stay. Of these, nine (81.8%) were diagnosed as superficial SSIs and two (18.2%) developed deep SSI infections.

### Patient demographics and baseline characteristics

The patients’ ages ranged from 16 to 47 years of age with a mean age of 31.2. The mean BMI of 799 patients was 31.9, with a range from 16.34 to 68.42 kg/m^2^. There were various indications for caesarean delivery among the study subjects; the most common one was a previous caesarean section (278; 34.4%), followed by failure to progress and abnormal position and presentation of the foetus, with 83 and 79 patients, respectively, representing almost the same percentages (10%). Further details regarding patient demographics and baseline characteristics are presented in Table1. In this study, a single dose of cefazolin antibiotic was the most frequently (100.0%) administered operative antibiotic prophylaxis (given to all studied mothers who underwent caesarean delivery across the participating hospital). The most frequent (100.0%) time of that antibiotic administration was within 1 h before skin incision. The mothers who had caesarean section were operated by multiple surgeon with similar qualifications (grade for all operators was consultant physician with almost same experience and outcome). The frequency of identified microorganisms is shown in Table 2.

**Table 1:** Univariate analysis of patient and maternal-related risk factors for SSI, post-caesarean section, throughout Al Ain Hospital, January 2016–December 2017.

Variable	SSI Absent (n = 796)	SSI Present (n = 11)	Total (n = 807)	P value
**Age, mean (SD), years**	31.3 (5.8)	30.7 (8.3)	31.2 (5.8)	0.673
**Age strata**^**a**^**, n (%), years**				0.441
< 35	555 (69.7)	7 (63.6)		
≥ 35	241 (30.3)	4 (36.4)		
**BMI, mean (SD)**	31.9 (5.7)	30.9 (5.7)	31.9 (5.7)	0.609
**BMI strata**^**b**^**, n (%)**				0.276
< 25	73 (9.3)	2 (18.2)		
≥ 25	715 (90.7)	9 (81.8)		
**Anesthesia type, n (%)**				0.926
Spinal	547 (68.7)	7 (63.6)		
General	232 (29.1)	4 (36.4)		
Epidural	14 (1.8)	0 (0.0)		
Epidural and general	3 (0.4)	0 (0.0)		
**Duration of Procedure, mean (SD), mints**	68.6 (27.5)	64.8 (19.0)	68.5 (27.4)	0.356
**Length of stay, mean (SD), days**	5.2 (4.2)	4.8 (1.6)	5.1 (4.1)	0.737
**Gestational age, mean (SD), Wks**	37.2 (2.8)	38.8 (1.8)	37.1 (2.8)	**0.041**
**Gestational age strata**^**c**^**, n (%)**				0.217
Preterm	354 (45.6)	1 (9.1)		
Early term	184 (23.7)	3 (27.3)		
Full term	193 (24.8)	4 (36.3)		
Late term	46 (5.9)	3 (27.3)		
**Duration of anesthesia, mean (SD), mints**	92.5 (48.2)	104.4 (23.0)	92.6 (48.0)	0.713
**Mode of delivery strata**^**d**^**, n (%)**				**0.092**
Emergency	632 (79.4)	11 (100.0)		
Elective	164 (20.6)	0 (0.0)		
**First CS strata, n (%)**				0.347
No	474 (62.5)	7 (77.8)		
Yes	284 (37.5)	2 (22.2)		
**Presence of diabetes strata, n (%)**				0.942
No	571 (71.7)	8 (72.7)		
Yes	225 (28.3)	3 (27.3)		

Variables with *P* values < 0.2 are presented in bold.

Body mass index (BMI): was calculated by dividing the woman’s weight (in kilograms) by height (in metres square).

^a^Stratified based on internationally standard categories ^45^.

^b^The classification of the World Health Organisation was employed in grouping BMI values ^45^.

^c^Gestational age was classified into groups depending on the age of a pregnancy in weeks starting from the last menstrual period of the women as following: Preterm (< 37 weeks); Early term (37–38 weeks); Full term (39–40 weeks); Late term (41–42 weeks).

^d^Mode of delivery is depending on the urgency, grade 1, 2 and 3 was classified as emergency, grade 4 and others classified as elective.

**Table 2:** Frequency of the microorganisms isolated from wound cultures.

Type of microorganism	Frequency (%)*
Gram negative bacilli-*Enterobacteriaceae*	36.4
*Escherichia* coli	9.1
*Proteus* species	9.1
*Klebsiell*a species	18.2
*Staphylococcus* species	36.4
*Streptococcus* species	9.1
*Streptococcus pyogenes* -Group A	9.1
Others	18.2

*Estimated based on a total of 11.

### Univariate analysis of risk factors for post-caesarean SSIs

Univariate analysis was done to find correlations between variables and SSIs during the 2 years. Table 1 shows the results of univariate analysis. There were individual relationships (with *P*-values < 0.2) between having an SSI following caesarean delivery and: non-categorized gestational age (*P* = 0.041) and mode of caesarean delivery (*P* = 0.092).

### Multivariate analysis of risk factors for post-caesarean SSIs

All variables that reached the selected significance level (i.e., with *P* values < 0.2 or that were clinically important) in the univariate analysis were included as explanatory variables in backward multivariable logistic regression modelling. The outcome variable, within this model, was the development of a post-caesarean SSI (yes/no).

The developed multivariate model (Table 3) highlights the variable that is an independent predictor for post-caesarean SSI: increasing gestational age. The analysis showed that the odds ratio of having post-caesarean SSI rises 1.5 fold each one-week increase in the gestational age of the foetus (95% CI 1.40–2.28, *P* = 0.045]. This explanatory model’s overall percentage for correct predictions of developing post-caesarean SSIs was found to be 91.0%.

**Table 3:** Multivariate logistic regression analysis of risk factors for post-caesarean SSI, throughout Al Ain Hospital, January 2016–December 2017.

Variable	Sub-group	Coefficient	*P* value	Odd ratio	95% CI Lower–Upper
**Age, year**		− 0.093	0.338	0.911	0.754–1.102
**Age strata**^**a**^**, year**					
	< 35			1	
	≥ 35	0.662	0.346	1.938	0.490–7.665
**BMI**		− 0.043	0.537	0.958	0.837–1.097
**BMI strata**^**b**^					
	< 25			1	
	≥ 25	− 0.357	0.781	0.699	0.056–8.683
**Anesthesia type**					
	Spinal			1	
	General	1.034	0.158	2.811	0.699–11.810
	Epidural	− 16.561	0.999	0.000	0.000–0.000
	Epidural & general	− 16.150	0.999	0.000	0.000–0.000
**Duration of procedure, mints**		− 1.714	0.192	0.180	0.014–2.359
**Length of stay, days**		− 0.003	0.983	0.977	0.726–1.367
**Gestational age, Wks**		0.409	**0.045**	1.506	1.400–2.283
**Gestational age strata**^**c**^					
	Preterm			**1**	
	Early term	1.076	0.488	2.932	0.141–61.143
	Full term	1.290	0.542	3.634	0.058–228.826
	Late term	1.462	0.623	4.316	0.013–1475.565
		0.008	0.283	1.008	0.944–1.023
**Mode of delivery strata**^**d**^					
	Emergency			**1**	
	Elective	− 17.203	0.995	0.000	0.000–0.000
**First CS strata**					
	No			1	
	Yes	− 1.009	0.227	0.365	0.071–1.871
**Presence of diabetes strata**					
	No			1	
	Yes	− 0.029	0.971	0.970	0.184–5.102

Variables with *P* values ≤ 0.05 are displayed in bold.

B: Coefficient.

Body mass index (BMI): was calculated by dividing the woman’s weight (in kilograms) by height (in metres square).

^a^Stratified based on internationally standard categories ^45^.

^b^The classification of the World Health Organisation was employed in grouping BMI values ^45^.

^c^Gestational age was classified into groups depending on the age of a pregnancy in weeks starting from the last menstrual period of the women as following: Preterm (< 37 weeks); Early term (37–38 weeks); Full term (39–40 weeks); Late term (41–42 weeks).

^d^Mode of delivery is depending on the urgency, grade 1, 2 and 3 was classified as emergency, grade 4 and others classified as elective.

## Discussion

In 1985, the World Health Organization (WHO) set an optimal rate of 10–15% for caesarean birth to optimize maternal and infant health, with the goal of minimizing the discrepancies in caesarean sections rates among countries^31^. The incidence of the caesarean section was found 17.4% in the present study. The caesarean section rate in the studied hospital is lower than average rate (33.0%) in the United Arab Emirates and lower than the global average rate^3,7,32^. Many authors believe that caesarean birth rate is affected by multiple contributing factors prevailing at individual, organizational and cultural levels^31^. Educating of pregnant women and of their physicians in order to enhance vaginal birth may have contributed to lower caesarean birth rate among the mothers studied. Further studies are needed to investigate factors affecting the choice of birthing process among pregnant mothers in the UAE. Additionally, the institution-specific factors should be taken into account for future studies to give a better understanding of these determinants.

The rate of delivery by caesarean sections is increasing globally and, therefore, the incidence of post-caesarean SSIs is probably also going to rise^2^. In the present study, 11 mothers developed SSIs among 807 caesarean sections, representing an SSI incidence of 1.4%. This translates to one in every 73 women who underwent caesarean section developing an SSI. In 2012, Gong et al. published a study of 8 Chinese centres from 2005–2009 that showed that of 13798 women who had a caesarean procedure, 96 cases developed an SSI, with a percentage of 0.7%^33^. Another study conducted in South Africa at an academic hospital found the percentage of post-caesarean SSIs to be 12.5%^34^. The SSI frequency following caesarean section was reported as 24.0% in a Tanzanian district hospital^35^. In our study, all SSI infections were diagnosed post hospital discharge, within 30 days after the date of the procedure. The short duration of hospital stays following caesarean sections can explain this. Previous studies have found that between 27.0% and 95.0% of all post-caesarean surgical site infections occur after discharge from hospital^36^. Charrier and colleagues reported that 85.0% of SSI infections developed after caesarean operations were observed after hospital discharge^37^. In a study conducted in southern Poland almost half (48.7%) of all SSIs were diagnosed prior to patients discharged from hospital^28^. This appeared to reflect relatively the effectiveness of active postdischarge detection of SSI in the present study.

In the present study, superficial infections constituted the majority of the SSIs, accounting for nine (81.8%), followed by deep surgical site infections, with two (18.2%). Superficial surgical site infections are less severe and can be treated by repeated dressing and providing broad-spectrum oral antibiotics. However, deep SSIs are more serious and can lead to long-term hospitalization and, in some cases, readmission. They also require repeated dressings, along with IV broad-spectrum antibiotics^20–23^.

Many studies have shown different percentages of SSIs, but all have reported that most SSIs are superficial infection^28^. Amenu et al. determined the rates and risk factors for obstetric cases and reported that 67.0% of SSI cases were superficial, 21.6% were deep infections, and 11.4% were organ/space infections^38^. Another multicentre study performed in three African countries found that 93.0% of SSIs were superficial^39^.

In such infections, the source of pathogens is usually the skin at the incision site (e.g., Staphylococcal or Streptococcal bacteria or a combination of aerobic and anaerobic bacteria). The dominance of gram-negative *Enterobacteriaceae* rods in our study was observed, which account for 36.4% of all detected pathogens; this may possibly due to inadequate obstetric hygiene among participated mothers. Similar microbiology pattern was found in a study conducted in polish hospitals where *Enterobacteriaceae* represented 50% of all isolated bacteria^28^.

Many risk factors have been identified for surgical site infectious morbidity after caesarean delivery. These represent about 10% of deaths associated with pregnancy^12,23–26,36,40,41^. The present study identified the increased gestational age of babies as a significant predictor for the development of SSIs after caesarean sections. Previous studies did not demonstrate an important association between gestational age and SSIs^33,42–44^. However, Wloch et al. showed that the risk of SSIs decreases at smaller gestational ages (< 37 weeks) and increases at a gestational age greater than 40 weeks^45^. In our study the increased likelihood of infection at greater gestational age could be explained by the assumed increase in the level of sleep disorders, anxiety and stress encountered, after caesarean sections, by the mothers who delivered full/late term infants. Anxiety and stress disorders lead to a delay in the healing of wounds as they have a harming impact on the immune system, which in turn enhances susceptibly to the development of wound infection^46^. A study conducted on 119,254 women who delivered following completion of the 37th week found that both maternal and neonatal risk increase if delivery occurred at the 40th week or beyond; such complications included chorioamnionitis and perineal laceration of 3rd and 4th degree^47^. The assumed occurrence of such maternal complications may have led to a new complication for such types of deliveries, that is wound infection as demonstrated in the present study. Additionally, this association may be due to the higher weight of a full-term foetus than a preterm foetus. Delivering a full term foetus may need longer/wider skin incision required to perform caesarean operation. Granulation tissue plays a key role in bringing the wound edges together (wound closure). This type of wound healing takes place in the case of chronic injuries (e.g. involving an open wound with dead spaces). This wound healing process may be prolonged in case of delivering full term foetus which may possibly have increased SSI incidence among the investigated mothers^46,48^.

To maintain a sufficient serum concentration of administered antibiotics during the surgical procedure, both the amended UK National Institute for Clinical Excellence guidelines (2011) and the Society of Obstetricians and Gynaecologists of Canada Infectious Diseases Committee (2010) recommend the administration of an adequate dose of an antibiotic agent within 60 min prior to skin incision^8,9,45,49^. NICE clinical guidelines (2011) recommended giving prophylactic antibiotics, such as a single dose of first-generation cephalosporin or ampicillin, to decrease the risk of post-operative SSI infections^8^. In this study, a single dose of cefazolin antibiotic was the most commonly administered operative antibiotic prophylaxis (delivered to all studied mothers who underwent caesarean delivery at the participating hospital). The most frequent (100.0%) time of administration was within 1 h before skin incision. These latter findings agree with the recommendation of NICE clinical guidelines (2011) regarding the best antibiotic selection and time to administer antibiotic prophylaxis. Of note, a previous study carried out during the period from 2013 to 2014 in Małopolska, Poland to evaluate data on outpatient antibiotic use in women post operation. The results showed that antibiotic use occurred significantly more commonly among those who underwent caesarean section (8.1%). The most frequently prescribed antibiotic was found β-lactams^50^.

The ECDC definitions were applied to identify post-caesarean SSIs after patient discharge, considering the time of symptom onset, e.g., symptoms identified during 30 days postoperatively. The cases of post-discharge, post-caesarean SSI were only registered during visits to the hospital outpatient clinic or readmission to obstetrics and gynaecology ward. No post-discharge surveillance was performed in the study hospital; however, the active SSI surveillance (applied in the current study in its present from) provides valuable information that can be used to improve care in this patient population. To monitor and manage any detected SSIs within 30-days post-discharge, follow-up period, the patients were routinely educated and instructed by hospital midwives (and patient counselling personnel) to judge the presence of SSIs and to arrange immediately for a confirmatory diagnostic examination at AAH. Additionally, as a part of routine practice, a nurse from the hospital infection control team was allocated to respond to any clarifications or queries requested regarding any wound signs of SSI appeared 30 days after operation date, as well as to arrange for a follow-up visit to the hospital outpatient clinic or for rehospitalisation within obstetrics and gynaecology ward (if required).

In conclusion, the present study measured for the first time at Emirati hospital the rate of SSI following caesarean sections within 30 days postoperatively and identified potential predictor for SSIs. Although identified variable is non-modifiable, that is larger gestational age, special measures can be adopted and applied at the clinical setting to provide optimal management and follow-up for this subpopulation. Another aspect of improvement is developing a post-discharge surveillance monitoring system across the UAE hospitals. However, employing multiple methods alternatively could also assist in identifying the largest possible proportions of these infections^18^. This could be achieved by developing a special information technology infrastructure to create a hospital database that automatically links different possible sources of SSIs identification, including microbiological data, inpatient wards, outpatient clinics as well as patient and therapy-related factors that could influence SSIs.

### Study strength and limitations

This is the first study conducted in the UAE evaluating the incidence of SSIs following caesarean sections. A strength of our study is that women were followed up for 30 days postoperatively. In addition, the CDC definition was applied to diagnose SSI following caesarean procedures; this can help benchmark post-caesarean SSIs in AAH and compare the hospital with other medical institutions worldwide. The study has the following limitations: (1) the retrospective nature of the study makes it susceptible to biases and confounding. Even though we controlled for confounding in the multivariate regression, we still cannot conclude causality of the identified risk factors for SSIs, as other confounding factors may exist. We can therefore only correlate the risks identified to the outcome variable (post-caesarean SSIs). Another constraint (2) is that we probably underestimated the true incidence of SSIs because some women with SSIs may have attended other hospitals (i.e., maybe due to presence of personal or economic reasons) in the 30-day follow-up period after the operation. Also, (3) it was not possible to account for some other important covariate variables, such as American Society of Anaesthesiologists (ASA) class, wound class and type of procedure performed. These factors may partially explain the variance that was unaccounted for in the logistic regression modelling. Finally, (4) the study was limited to one hospital; thus, the results on the incidence of SSI infection might not be generalizable. However, these limitations reveal more opportunities for further research on the risk analysis of SSIs after caesarean sections in the UAE.

## Conclusions

The incidence rate of post-caesarean section SSIs in the study site hospital was found to be 1.4%. The variable of increased gestational age was found to be an independent predictor for post-caesarean SSIs.

## Data Availability

The datasets generated during the current study are available from the corresponding author on reasonable request.
